# A screen for Fli-1 transcriptional modulators identifies PKC agonists that induce erythroid to megakaryocytic differentiation and suppress leukemogenesis

**DOI:** 10.18632/oncotarget.14377

**Published:** 2016-12-30

**Authors:** Tangjingjun Liu, Yao Yao, Gang Zhang, Ye Wang, Bin Deng, Jialei Song, Xiaogang Li, Fei Han, Xiao Xiao, Jue Yang, Lei Xia, You-Jun Li, Maksym Plachynta, Mu Zhang, Chen Yan, Shuzhen Mu, Heng Luo, Eldad Zacksenhaus, Xiaojiang Hao, Yaacov Ben-David

**Affiliations:** ^1^ Department of Biology and Chemistry, The Key Laboratory of Chemistry for Natural Products of Guizhou Province and Chinese Academy of Sciences, Guizhou, China; ^2^ College of Ecology, Lishui University, Zhejiang, China; ^3^ School of Pharmaceutical Sciences, Guizhou University, Guizhou, China; ^4^ Department of Anatomy, Norman Bethune College of Medicine, Jilin University, Changchun, China; ^5^ Department of Medical Biophysics, University of Toronto, Toronto, Ontario, Canada; ^6^ Division of Advanced Diagnostics, Toronto General Research Institute–University Health Network, Toronto, Ontario, Canada; ^7^ State Key Laboratory of Functions and Applications of Medicinal Plants, Guizhou Medical University, Guiyang, China; ^8^ The Laboratory of Cell Biochemistry and Topogenic Regulation, College of Bioengineering and Faculty of Sciences, Chongqing University, Chongqing, China

**Keywords:** Fli-1, PKC, erythroid and megakaryocytic differentiation, leukemia therapy, drug screens

## Abstract

The ETS-related transcription factor Fli-1 affects many developmental programs including erythroid and megakaryocytic differentiation, and is frequently de-regulated in cancer. Fli-1 was initially isolated following retrovirus insertional mutagenesis screens for leukemic initiator genes, and accordingly, inhibition of this transcription factor can suppress leukemia through induction of erythroid differentiation. To search for modulators of Fli-1, we hereby performed repurposing drug screens with compounds isolated from Chinese medicinal plants. We identified agents that can transcriptionally activate or inhibit a Fli-1 reporter. Remarkably, agents that increased Fli-1 transcriptional activity conferred a strong anti-cancer activity upon Fli-1-expressing leukemic cells in culture. As opposed to drugs that suppress Fli1 activity and lead to erythroid differentiation, growth suppression by these new Fli-1 transactivating compounds involved erythroid to megakaryocytic conversion (EMC). The identified compounds are structurally related to diterpene family of small molecules, which are known agonists of protein kinase C (PKC). In accordance, these PKC agonists (PKCAs) induced PKC phosphorylation leading to activation of the mitogen-activated protein kinase (MAPK) pathway, increased cell attachment and EMC, whereas pharmacological inhibition of PKC or MAPK diminished the effect of our PKCAs. Moreover, in a mouse model of leukemia initiated by Fli-1 activation, the PKCA compounds exhibited strong anti-cancer activity, which was accompanied by increased presence of CD41/CD61 positive megakaryocytic cells in leukemic spleens. Thus, PKC agonists offer a novel approach to combat Fli-1-induced leukemia, and possibly other cancers,by inducing EMC in part through over-activation of the PKC-MAPK-Fli-1 pathway.

## INTRODUCTION

Transcription factors (TFs) are frequently modulated during cancer initiation and progression [1, 2]. Despite difficulties in targeting TFs for inhibition, new strategies including siRNA/miRNA knockdown, disruption of protein-protein interaction and modulation of chromatin accessibility are being developed to meet this challenge [1]. Members of ETS family of TFs are often altered in various cancers, including leukemia [2]. The Friend Leukemia virus integration-1 (Fli-1), a member of ETS gene family, is activated by retroviral insertional mutagenesis in F-MuLV-induced erythroleukemias [3, 4]. This TF also undergoes translocation in most human Ewing sarcomas, generating an EWS-Fli-1 fusion protein with potent oncogenic activity [5]. Fli-1 activation affects several hallmarks of cancer including proliferation, apoptosis, differentiation, angiogenesis, genomic instability and immune function [2]. Fli-1 expression is induced in various human cancers including breast, melanoma, lymphomas and leukemia [6–11]. In breast cancer, Fli-1 expression is correlated with tumorigenesis, invasion and metastasis [12, 13]. In addition to cancer, Fli-1 affects various human autoimmune diseases including Systemic Lupus Erythematosus (SLE) and Systemic sclerosis/scleroderma [14–18]. Based on these diverse biological properties, Fli-1 may be an excellent therapeutic target for various diseases and cancers that are driven by high levels of this ETS factor.

In the past decade, several strategies have been employed to isolate inhibitors for Fli-1 or EWS-Fli-1. These efforts resulted in identification of small molecules targeting DNA - or RNA-binding activity of Fli-1 [19–22]. Our group has used a luciferase-based expression assay to identify antagonists of Fli-1, some of which displayed strong anti-cancer activity in culture and animal models of leukemia [23]. Among these agents was a class of anti-Fli-1 compounds that altered calcium uptake and inhibited protein kinase C-delta (PKCδ). These PKCδ inhibitors blocked the phosphorylation of Fli-1, an event that is a critical for inducing its DNA binding activity [23, 24]. Unfortunately, these anti-Fli-1 compounds bind other targets in addition to PKCδ, and, there is therefore a pressing need to identify specific inhibitors of Fli-1. Here we describe a repurposing drug screen with Chinese medicinal compounds, and the identification and characterization of potent Fli-1 activating compounds. Remarkably, we show that induction of Fli-1 via these compounds induce erythroid to megakaryocytic differentiation (EMD) via PKCγ and suppress leukemogenesis both *in vitro* and *in vivo*.

## RESULTS

### Identification of Fli-1 transcriptional activating compounds

**Figure 1:**
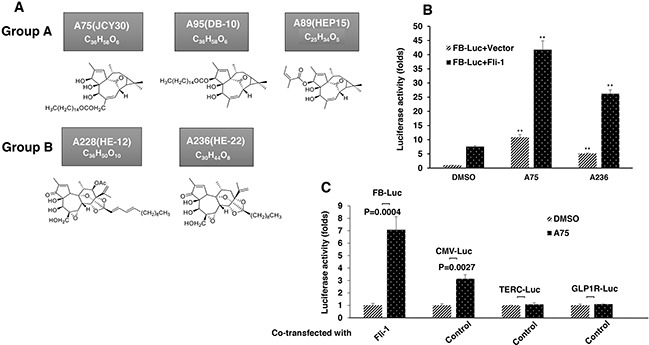
Identification of Fli-1 transcription activating compounds **A**. Structure and chemical composition of Group A and B compounds. **B**. A75 and A236 compounds increase transcriptional activity of FB-Luc (1.25 μg) reporter gene co-transfected with MigR1-Fli-1 (1.25 μg) or MigR1 (1.25 μg) vectors into HEK293T cells. **C**. A75 and A236 compounds moderately increase luciferase activity of control CMV-Luc but not of TERC-Luc or GLP1R-Luc promoters. Compound concentration in panels B-C, 1μM. P values calculated by student t-test. P< 0.005 denoted by **.

To identify Fli-1 modulators, we performed a repurposing drug screen using a luciferase-based assay with a reporter plasmid, FB-Luc, in which two consensus Fli-1 binding sites are inserted upstream of a minimal promoter [23]. About 1500 compounds from Chinese medicinal plants and their derivatives were tested for induction or repression of luciferase activity using high content screen in 96 well plates. Several compounds capable of activating or inactivating Fli-1 transcriptional activity of FB-Luc have been identified. Like our previous study [23], the anti-Fli-1 inhibitors induced erythroid differentiation and suppressed growth (unpublished results). Here we focused on 5 compounds, structurally classified into two groups (Figure 1A), which strongly increase Fli-1 transcriptional activity. Representatives of group A (A75 = JCY30) and group B (A236 = HE-22) increased luciferase activity of the Fli-1 reporter plasmid around seven fold when co-transfected together with MigR1-Fli-1 expression vector into HEK293T cells (Figure 1B). These two compounds increased luciferase activity of control plasmid driven by the CMV promoter (CMV-Luc) three folds, but had no effect on control TERC and GLP-1R promoter constructs (Figure 1C). Other compounds identified by our screen exhibited Fli-1 transactivating activity similar to A75 and A236 (data not shown).

### Fli-1 transactivating compounds exhibit biological properties similar to the phorbol ester compound TPA

**Figure 2:**
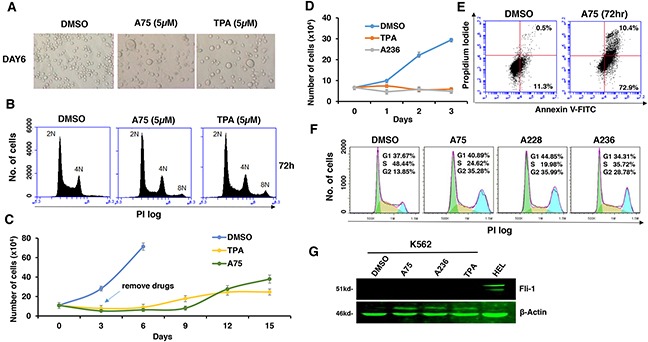
Fli-1 transactivating compounds block proliferation, and induce polyploidy as well as attachment of Fli-1- expressing erythroleukemic cells in culture **A**. Micrographs showingA75 and TPA increase the attachment and appearance of large multi-nuclear cells in CB7 cell line, 3 days post-drug incubation (magnification x40). **B**. CB7 cells treated with A75 or TPA exhibit increased polyploidy associated with appearance of 2N, 4N and 8N chromosomes, 72 hours post treatment. **C**. A75 and TPA strongly suppress growth of CB7 cells, even when the compounds are removed 3 days post drug treatment. **D**. A236 and TPA suppress growth of K562 cells in culture. **E**. A75 treatment increases the percentage of Annexin V-positive apoptotic cells in K562 cells 72 hours post drug treatment. **F**. A75, A228 and A236 increase G2/M and decrease S phase stages of cell cycle in K562 cells, 16 hour post-drug incubation. Compound Concentration in panels A-F, 5μM. **G**. Indicated compounds (2μM) did not induce Fli-1 expression in K562 cells, as determined by Western blot analysis. HEL cells were used as positive control.

Fli-1 transactivating compounds are structurally related to diterpenes, a family of drugs including 12-O-tetradecanoylphorbol-13-acetate (TPA), which promotes tumorigenesis when applied to the skin [25, 26]. To determine their biological activity, leukemic cell lines expressing high or low levels of Fli-1 were treated with the compounds. Treatment of murine erythroleukemia cell line CB7 with A75 increased cell size and attachment (Figure 2A). Cell enlargement was accompanied by polyploidy with appearance of N2, N4 and N8 chromosomes (Figure 2B). Similar characteristics were previously reported in erythroleukemic cells treated with TPA, as demonstrated in Figure 2A and 2B [27]. Growth inhibition by both A75 and TPA was long lasting even when these agents were removed from the culture three days post treatment (Figure 2C). A similar trend of cell attachment, polyploidy and growth inhibition was observed in the human erythroleukemia cell line HEL (not shown). Other compounds listed in Figure 1A exhibited similar growth inhibition as A75 (data not shown).

**Figure 3:**
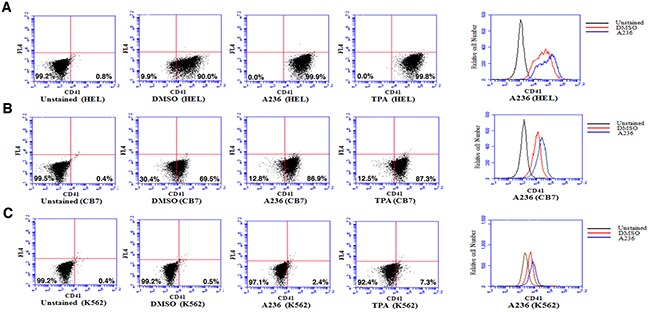
Fli-1 transactivating compounds induce megakaryocytic differentiation in erythroleukemia cell lines Treatment of the erythroleukemic cell lines HEL **A**. and CB7 **B**. with A236 and TPA increases the percentage and level of CD41 expression, 48 hours post- treatment. **C**. These compounds only weakly induce CD41 expression in K562 cells. Compound concentration, 5μM.

**Figure 4:**
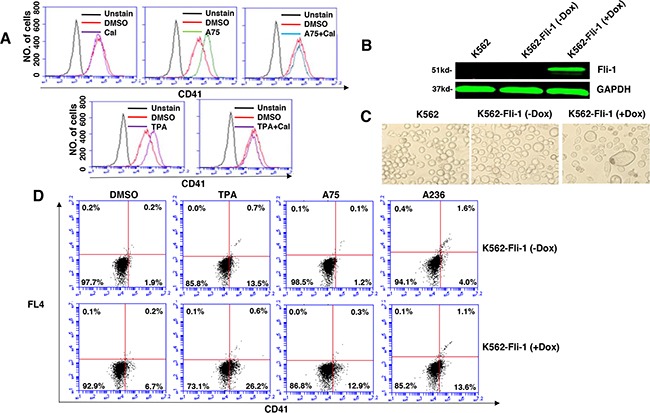
Induction of Fli-1 expression promotes megakaryocytic differentiation **A**. The A75- and TPA- induced CD41 expression in HEL cells can be blocked by treatment with Calcimycin (5μM). **B**. Western blot of K562 and K562-Fli-1^indu-20-on^ cells treated or not with Doxycycline (DOX) for, 24 hours. **C**. Induction of Fli-1 in K562-Fli-1 cells by DOX (1μM) increases the number of large, polynucleated cells compared to control cells. **D**. Induction of CD41 expressing cells in K562-Fli-1 cells with or without DOX treatment plus the indicated compounds.

Like CB7 and HEL, the human erythroleukemia cell line K562, which does not express detectable levels of Fli-1, ceased proliferation in culture after treatment with A75 or TPA (Figure 2D). However, unlike CB7 and HEL, the majority of K562 cells underwent apoptosis shortly after treatment (Figure 2E), which was associated with G2M cell cycle arrest (Figure 2F). Notably, Fli-1 expression was not induced in K562 cells by these compounds (Figure 2G). This is in contrast to the effect of TPA, which was reported to induce Fli-1 expression in these cells [28–30]. While both Fli-1 expressing CB7 and HEL cells treated with A236 and TPA compounds underwent differentiation to express high levels of the megakaryocyte marker CD41, K562 cells were unaffected compared to control DMSO-treated cells (Figure 3A–3C). Similar results were observed with K562 cells obtained from two different sources/countries. In HEL cells, the increased intensity of CD41 expression induced by the A75 and TPA compounds can be blocked by treatment with the previously identified Fli-1 inhibitor Calcimycin [23](Figure 4A).

To further assess the effect of Fli-1 expression on K562 cell differentiation, we used a doxycycline (DOX) inducible system, which enabled us to overcome the cell death induced by Fli-1 in these cells. Dox-dependent induction of Fli-1 in K562-Fli-1^indu-20-on^ cells (Figure 4B) resulted in appearance of large, polynucleated cells (Figure 4C). Remarkably, Fli-1 expression in these cells induced CD41 levels (Figure 4D). CD41 expression was further elevated when K562-Fli-1^indu-20-on^ cells were treated with both DOX and A75, A236 and TPA compounds (Figure 4D). Overall, these results demonstrate that while Fli-1 is critical for megakaryocyte differentiation of K562 cells, in the absence of this TF, these compounds alter other pathway(s) to promote apoptosis.

### Fli-1 transactivating compounds signal through the MAPK pathway

**Figure 5:**
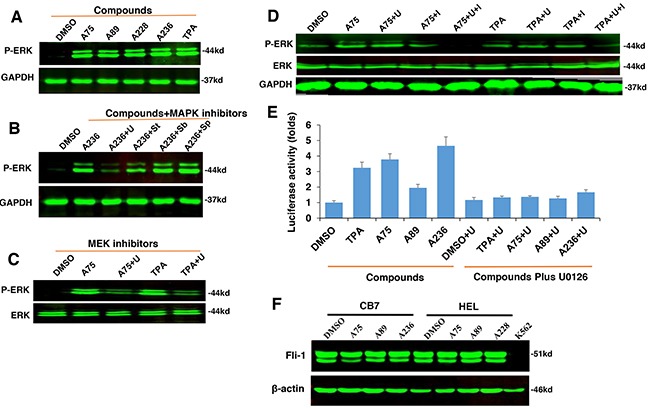
The compounds induces Fli-1 transcriptional activity through MAPK/ERK **A**. Indicated compounds induce phosphorylation of MAPK/ERK in CB7 cells. **B**. Induction of MAPK/ERK by A236 can be blocked by the MEK inhibitor U0126 (U). Phosphorylation was not blocked by JNK SP600125 (Sp) or P38 SB203580 (Sb) inhibitors, respectively, but partially by the RAF inhibitor Sorafenib tosylate (St). **C**. A75- and TPA-induced phosphorylation of MAPK/ERK is inhibited by U0126 in HEL cells. **D**. Induction of MAPK/ERK phosphorylation by A75 and TPA is blocked by addition of both U0126 and BCR-ABL inhibitor Imatinib in K562 cells, **E**. Fli-1 transcriptional activity induced by the indicated compounds in HEK293T cells co-transfected with FB-Luc+MigR1-Fli-1 is blocked by MEK inhibitor U0126. **F**. Western blot analysis for Fli-1 expression in CB7 and HEL cells after treatment with A75, A89 and A228. Compound concentration in experiments A-F, 2μM. Concentration of MAPK/ERK inhibitors, 5μM.

Since TPA is a known inducer of mitogen-activated protein kinase (MAPK) [31], we tested our compounds for induction of MAPK phosphorylation. In CB7 cells treated with several Fli-1 transactivating compounds, we observed rapid phosphorylation of MAPK/ERK (Figure 5A). Similar to TPA, ERK phosphorylation was blocked by treatment with the MEK inhibitor U0126 (U) and partially by the RAF inhibitor Sorafenib (St), but not with the JNK or p38 inhibitors SP600125 and SB203580, respectively (Figure 5B). Similar MAPK phosphorylation by these compounds and inhibition by U0126 were observed in the erythroleukemia cell line HEL (Figure 5C). In K562 cells carrying a BCR-ABL translocation, treatment with U0126 was not sufficient to block MAPK/ERK phosphorylation induced by A75 and TPA compounds. Addition of both U0126 and the BCR-ABL inhibitor Imatinib was required to block this phosphorylation (Figure 5D). Thus, Fli-1 transactivating compounds may cooperate with Imatinib for the treatment of chronic myelogenous leukemia (CML) driven by BCR-ABL.

Finally, we examined whether Fli-1 activation by these agents is mediated by MAPK/ERK phosphorylation. Transient transfection of FB-Luc plasmid with MigR1-Fli-1 expression vector into HEK293T cells resulted in several fold increase in luciferase activity after treatment for 12 hours with A236, A75, A89 and TPA compounds, and this was blocked by the MEK inhibitor U0126 (Figure 5E). Again, the JNK or p38 inhibitors SP600125 and SB203580 had no effect on this transcriptional activity (data not shown), pointing to MAPK/ERK as the downstream effector of Fli-1 transcriptional activation by these compounds.

### Differentiation induced by Fli-1 transactivating compounds is mediated through suppression of erythroid and induction of megakaryocytic target genes

Treatment of erythroleukemia cell lines CB7 and HEL with A75, A89 and A228 compounds slightly decrease the level of Fli-1 as measured by Western blots (Figure 5F). This suggests that these compounds exert their effect through a post-translational modification, as previously seen with anti-Fli-1 compounds [23].

**Figure 6:**
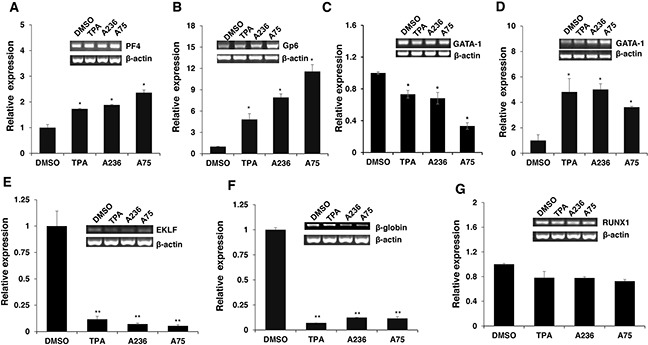
Fli-1 transactivating compounds alter expression of marker genes involved in erythroid to megakaryocytic differentiation Expression of marker genes for erythroid and megakaryocytic lineages PF4 **A**., GP6 **B**., GATA1/2μM **C**., GATA1/5μM **D**., EKLF **E**., β-globin **F**. and RUNX1 **G**. in Fli-1 expressing HEL cells treated for 24 hours with TPA, A236 and A75, using RT-PCR (inserts) and Q-RT-PCR (graphs). Compound concentrations for experiments in panel A-F, 5μM. All Q-RT-PCRs were performed in triplicates and repeated at least 2 times. P< 0.05 denoted by *; P< 0.005 by **.

**Figure 7:**
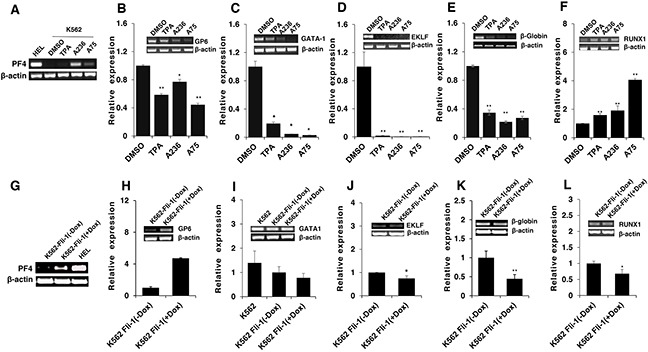
Induction of erythroid to megakaryocytic differentiation by compounds requires Fli-1 Expression of markers of erythroid and megakaryocytic lineages PF4 **A**., GP6 **B**., GATA1 **C**., EKLF **D**., β-globin **E**. and RUNX1 **F**. in Fli-1-negative K562 cells treated for 24 hours with or without TPA, A236 and A75 using RT-PCR (inserts) and Q-RT-PCR (graphs). Similar analysis in K562-Fli-1 cells with or without doxycycline (DOX) treatment for PF4 **G**., GP6 **H**., GATA1 **I**., EKLF **J**., β-globin **K**. and RUNX1 **L**., as indicated. Compound concentration, 5μM. Since PF4 expression is negligible in K562, HEL cells were used as a positive control (Panel A). All Q-RT-PCRs were performed in triplicates and repeated at least 2 times. P< 0.05 denoted by *; P< 0.005 by **.

To address the consequences of Fli-1 activation, expression of several Fli-1 target genes was determined using RT-PCR and q-RT-PCR. Specifically, we examined expression of megakaryocytic markers glycoprotein VI (GP6) and platelet factor 4 (PF4), both known to be positively regulated by Fli-1 [32–34]. As expected, mRNA levels of PF4 and GP6 were significantly elevated in HEL cells treated with TPA, A236 and A75 compounds (Figure 6A and 6B). Similar upregulation was detected after Fli-1 expression in CB7 cells (data not shown). In K562 cells lacking Fli-1, expression of PF4 was slightly induced by A75 and A236, but GP6 transcription downregulated by these agents (Figure 7A and 7B). However, induction of Fli-1 in K562-Fli-1 cells via DOX resulted in robust upregulation of PF4 and GP6 transcripts (Figure 7G and 7H). This result and appearance of CD41+ K562 cells induced by Fli-1 further support the role of this TF in megakaryocytic differentiation (Figure 4D). We also examined the expression of Fli-1 target gene GATA1 whose transcription is negatively regulated by Fli-1 and is necessary for both erythroid and megakaryocytic differentiation [29, 35, 36]. In HEL cells, TPA, A236 and A75 at 2μM significantly downregulated GATA1 mRNA (Figure 6C), but higher expression of this TF was detected at 5μM of these drugs (Figure 6D). In K562 cells, these compounds strongly downregulated GATA1 expression (Figure 7C). The Fli-1 induction in K562 cells led to suppression of GATA1, but this drop in expression was moderate compared with levels seen in the drug-treated K562 cells (Figure 7C and 7I). These results suggest that while the compounds downregulated GATA1 at lower concentration through a Fli-1-dependent mechanism, this suppression was inhibited at higher concentration of drugs, allowing this TF to promote megakaryocytic differentiation.

Next, we examined the effect of the Fli-1 transactivating compounds on erythroid differentiation. The transcription factor EKLF is directly suppressed by Fli-1 [37–39], whereas globin gene expression in indirectly regulated by this TF [4]. We found expression of both EKLF and globin was downregulated in HEL cells (Figure 6E and 6F) as well as in K562 cells (Figure 7D and 7E), 3 day post compound treatment. In K562-Fli-1^indu-20-on^ cells treated with DOX, EKLF and globin gene expression was suppressed, albeit more moderately (Figure 7J and 7K). Together, these results demonstrate that the compounds induce a switch from erythroid- to megakaryocytic-gene expression program in erythroleukemia cells.

RUNX1 plays a crucial role in adult (definitive) hematopoiesis [40]. It directly binds Fli-1 and other TFs to regulate EMD [41]. In HEL cells, RUNX1 expression was marginally changed in response to TPA, A236 and A75 compounds (Figure 6G), but significantly upregulated in drug-treated K562 cells (Figure 7F). Expression of Fli-1 in K562 cells moderately yet significantly reduced RUNX1 expression (Figure 7L). Thus, the compounds, but not Fli-1 alone, regulate the expression of RUNX1.

### Tumor inhibition by Fli-1 transactivating compounds is mediated through phosphorylation and activation of protein kinase C (PKC)

**Figure 8:**
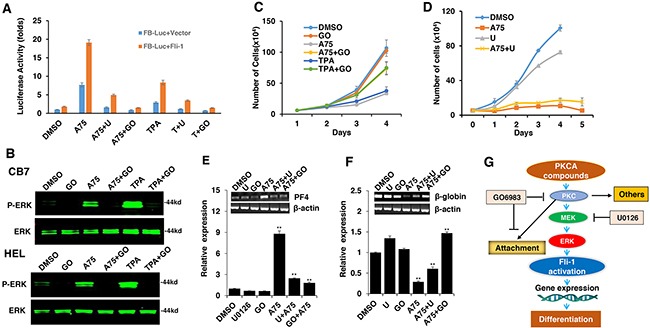
The Fli-1 transactivating compounds induce growth arrest and differentiation of erythroleukemic cells through PKC and, in part, through MAPK/ERK activation **A**. HEK293T cells co-transfected with FB-Luc and MigR1-Fli-1, and treated with A75 or TPA (1μM), show induction of Fli-1 transcriptional activity that can be blocked by U1026 (5μM) or GO6982 (2μM). **B**. Treatment of HEL or CB7 cells with A75 or TPA (2μM) induces MAPK/ERK phosphorylation that can be suppressed by GO6982 (2μM). **C**. Growth suppression induced by A75 or TPA (2μM) in HEL cells is reversed by GO6983 (2μM). **D**. Growth suppression induced in CB7 cells by A75 (2μM) is only marginally inhibited by U1026 (10μM). **E, F**. In CB7 cells, induction of megakaryocytic marker PF4 by A75 is completely inhibited by U0126 and GO6983 (5μM); these inhibitors suppressed downregulation of erythroid β-globin by A75. **G**. A model depicting the sequential events that occur in leukemic cells after treatment with PKCA compounds leading to Fli-1 transcriptional activation. PKCA compounds activate Fli-1 and also increase cell attachment independently of MAPK/ERK activation. PKCA compounds likely activate other pathways that further increase their growth inhibitory activity.

**Figure 9:**
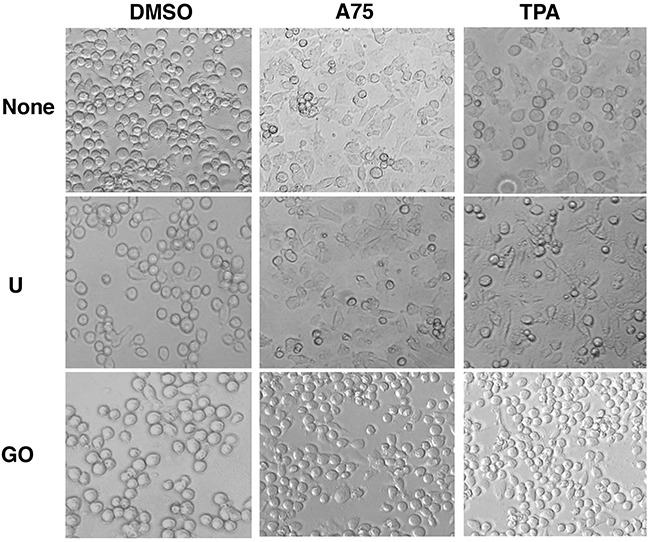
PKCA compounds induce cell attachment independently of MAPK/ERK activation CB7 cells were cultured in the presence of A75 and TPA compounds (2μM) with or without MEK U0126 (5μM) or PKC inhibitors GO6983 (2μM), respectively, as indicated. Cells were photographed 24 hours after treatments (magnification x20).

TPA and other phorbol ester compounds activate protein kinase C by mimicking diacyl glycerol (DAG), its natural agonist [31]. Previous studies by our group and others have demonstrated the importance of PKCγ phosphorylation during Fli-1 transcriptional activation [2, 23]. In accordance, using our luciferase assay, we found that Fli-1 mediated transcriptional activation by A75 and TPA could be inhibited by the PKC inhibitor GO6983 (Figure 8A). This inhibitor completely blocked MAPK phosphorylation in both HEL and CB7 cells even after treating with these compounds (Figure 8B). Growth inhibition induced by these compounds was also diminished by GO6983 (Figure 8C). The MEK inhibitor U0126 significantly inhibited CB7 cell growth, but only marginally rescued growth suppression induced by A75 (Figure 8D). Finally, both U0126 and GO6983 treatment significantly abolished the A75-mediated upregulation of PF4 and downregulation of β-globin genes in HEL cells, respectively (Figure 8E and 8F). These results demonstrate a critical role of Fli-1 and its upstream regulators PKC and MAPK/ERK in EMD. Thus, we designated our compounds as PKC agonists (PKCAs). While GO6983 blocked cell attachment induced by A75 and TPA, the MEK inhibitor did not (Figure 9). These results place PKC upstream of the MEK/Fli-1 differentiation pathway and suggest an additional role for PKC in cell attachment, which is independent of Fli-1 (Figure 8G).

**Figure 10:**
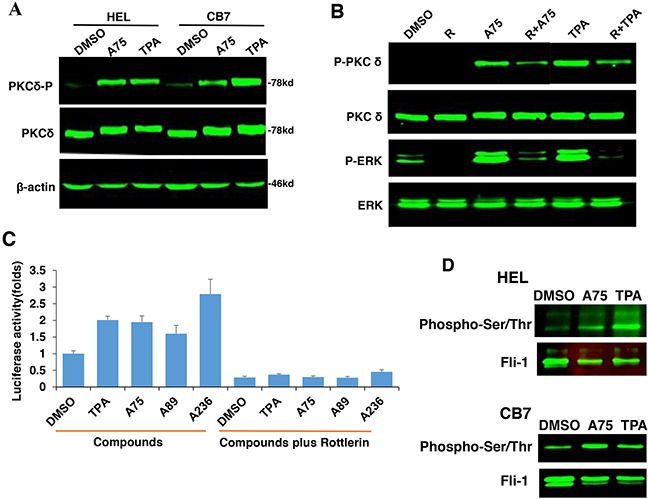
PKCA-induced phosphorylation of PKCδ, MAPK/ERK and induction of Fli-1 transcriptional activation can be reversed by the PKCδ-inhibitor Rottlerin **A**. A75 or TPA treatment (2μM) for 24 hours induces phosphorylation of PKCδ in both HEL and CB7 cell lines. **B**. Phosphorylation of MAPK/ERK and PKCδ in HEL cells treated with indicated compounds with or without PKCδ inhibitor Rottlerin. **C**. Fli-1 transcriptional activity induced by A75 and TPA compounds in HEK293T cells co-transfected with FB-Luc+MigR1-Fli-1 is blocked by Rottlerin. **D**. Extracts from CB7 and HEL cells were immunoprecipitated using anti-Fli-1 antibody followed by immune-blotting with anti-phospho-Serine/Threonine or anti-Fli-1 antibodies.

PKCAs strongly increased PKCδ phosphorylation in HEL and CB7 cell lines (Figure 10A), but had no effect on PKCα and nPKC-eta activities (data not shown). Treatment with the specific PKCδ-inhibitor Rottlerin [42, 43], reduced phosphorylation of both PKCδ and MAPK/ERK (Figure 10B), and completely blocked Fli-1 transactivation induced by Fli-1 or Fli-1+ indicated compounds (Figure 10C). Treatment of HEL cells with Rottlerin, similar to U0126 (Figure 9), did not block cell attachment, indicating involvement of other PKCs in this process (Data not shown). Interestingly, the PKCAs also increased phosphorylation of Fli-1 in HEL and CB7 cells, as detected using an anti-Serine/Threonine antibody (Figure 10D). This is in accordance with our previous work showing that Fli-1 inhibitors reduce this phosphorylation [23]. Together, these results demonstrate the importance of PKCδ in growth suppression downstream of our compounds.

### PKCA compounds induce megakaryocytic differentiation and suppress erythroleukemia in vivo

**Figure 11:**
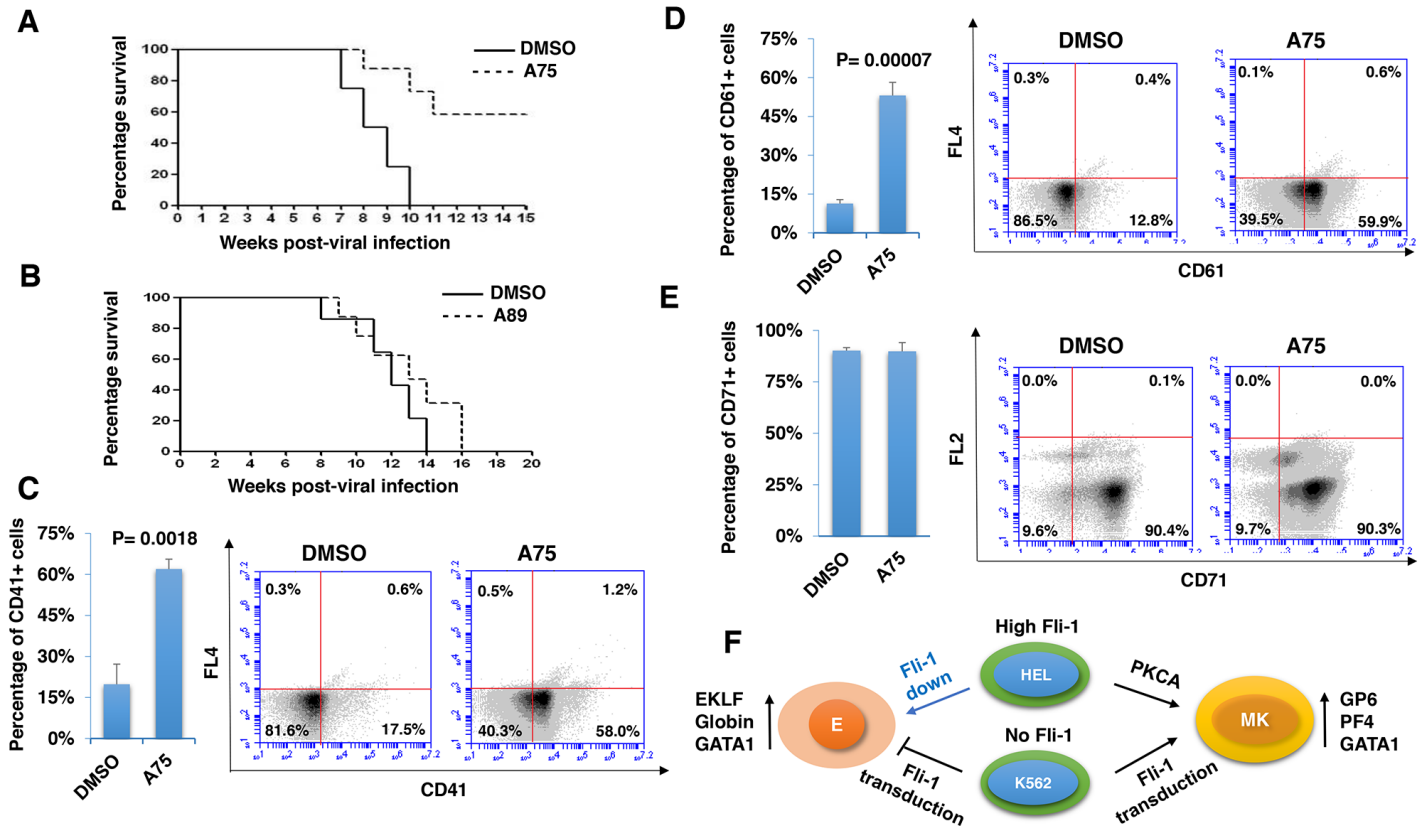
Distinct PKCA compounds can inhibit leukemogenesis in a mouse model of leukemia induced by Fli-1 retroviral insertional activation **A, B**. Groups of BALB/c mice (n=7) were infected at birth with F-MuLV and 5 weeks later treated with A75 or A89 compounds (3 mg/ kg body weight), every other day for a total of six injections. Latency to death was used to plot a Kaplan-Meire survival curve. For the A75-treated group (A), the experiment was terminated around 20 week post-viral injection. Splenocytes isolated from large spleens of leukemic mice were used to determine the percentage of cells expressing megakaryocytic CD41 **C**. and CD61 **D**., or erythroid CD71 **E**. markers, using flow cytometry. Individual and average flow charts for each experiment is shown. **F**. A model for PKCA drug-induced erythroid-to-megakaryocytic differentiation in erythroleukemia. In HEL cells, Fli-1 downregulation (Fli-1 down) through either siRNA [6] or pharmacological drugs [23] leads to erythroid differentiation. Conversely, Fli-1 activation by these compounds promotes megakaryocytic differentiation. In K562 cells which express no Fli1, Fli-1 transduction (Fli-1 up) also induces megakaryocytic differentiation. This model suggests that both Fli-1 inactivation and activation suppress leukemia either through erythroid or megakaryocytic differentiation, respectively. E - erythrocytes; MK - megakaryocytes.

To determine whether our PKCA compounds can suppress leukemia progression *in vivo*, we used a mouse model of erythroleukemias induced by F-MuLV, in which insertional activation of Fli-1 occurs as early as 40 days post viral injection [44], is the initial event during leukemogenesis [4]. We found that treatment of F-MuLV-infected mice with A75 significantly inhibited erythroleukemia development (Figure 11A). Consistent with its lower Fli-1 transcriptional activation (Figure 5E), A89 only weakly suppressed tumorigenesis in these mice (Figure 11B). In contrast to the above drugs, A236 demonstrated some toxicity and was therefore excluded from the *in vivo* analysis. Overall, our results demonstrate that some of our PKCAs, such as A75, can be utilized for the treatment of leukemia carrying an activated Fli-1.

We next sought to determine whether EMD observed *in vitro* also occurred *in vivo*. Since F-MuLV-induced leukemic cells mainly infiltrate the spleens, resulting in splenomegaly [4], tumors with similar volume were subjected to flow cytometry analysis. A higher number of splenocytes expressing megakaryocyte-specific markers CD41 and CD61 (59%-58%) were detected in leukemic spleens of A75-treated mice compared with DMSO-injected controls (12%-17%) (Figure 11C and 11D). In contrast, no significant change in the percentage of cells expressing the erythroid marker CD71 was observed (Figure 11E). In this case, leukemic cells that underwent EMC may also express CD71 [45]. As the majority of splenic cells represent erythroleukemia in these mice, the results suggest that over-activation of Fli-1 or activation of the PKC-MAPK-Fli-1 axis by A75 suppress leukemogenesis at least in part by inducing EMD.

## DISCUSSION

The ETS gene Fli-1 is a critical regulator of development and homeostasis. Over-expression of Fli-1 affects several hallmarks of cancer including proliferation, differentiation, apoptosis, angiogenesis and genomic stability [2]. Based on these abilities, much effort has been invested in the development of anti-Fli-1 drugs for the treatment of cancers driven by this TF. In this study, repurposing screens of compounds isolated from Chinese medicinal plants led to the identification of a group of compounds that induce Fli-1 transcriptional activity. Unexpectedly we found that these compounds suppressed, rather than induced, erythroleukemia both *in vitro* and *in vivo*. We demonstrate that these compounds, through regulation of several Fli-1 downstream target genes, promote megakaryocytic differentiation of leukemic cells, leading to growth inhibition. Thus, both inhibition and activation of Fli-1 appear to have therapeutic applications for leukemia.

Since Fli-1 was originally identified as an oncogene involved in erythroleukemogenesis, this raises the question of how drug-mediated over-activation of this proto-oncogene can reverse its function to become a tumor suppressor. As shown here and in other studies, Fli-1 expression is high in some erythroleukemia cell lines (HEL, CB7), but negligible in others (K562, KH16) [46]. While siRNA- or drug-mediated downregulation of Fli-1 in erythroleukemic cells with high Fli-1 expression induces erythroid differentiation [6], its activation by our PKCA compounds or its re-expression in Fli-1-negative cells specifically promotes megakaryocytic differentiation. These results suggest that although a certain level of Fli-1 can be tolerated in a given erythroleukemic cell to sustain proliferation, suppression of Fli-1 or further activation of this TF can induce differentiation to erythroid or megakaryocytic lineage, respectively, leading in both cases to cessation of growth (Figure 11F). This Fli-1 mediated restriction phenomenon is not limited to erythroleukemias and may also apply to other cancer types. For example, both high and low levels of Fli-1 were found to be associated with poor survival of human acute myelogenous leukemia (AML) [7]. In this case, Fli-1 inactivation in high-expressing AML cells should have therapeutic benefit as we showed for erythroleukemia [6, 23]. In low- and high-expressing AMLs with poor prognosis, altering Fli-1 threshold through forced expression could also negatively impact leukemic growth, as we have shown here for K562 and HEL cells, respectively. This restriction phenomenon may also be relevant to other transcription factors including E2F1: for instance, both up- and down-regulation of E2F1 were reported to have a negative effect on cell growth [47, 48].

Our results show that post-translational modification of Fli-1 is responsible for PKCA-induced megakaryocytic differentiation. PKCδ phosphorylation was critical for Fli-1 transcriptional activation as its pharmacological inhibition abolished growth inhibition and differentiation. Fli-1 has been shown to interact with RUNX1, and TPA reduces phosphorylation of serine-10 residue in Fli-1 that was found to be critical for this complex to promote megakaryocytic differentiation [49]. The importance of Fli-1 - RUNX1 interaction in megakaryocytic differentiation is also supported by the identification of inactivating mutations in both TFs in patients with excessive bleeding [50]. How these two TFs function and whether PKC is involved in this process are yet to be determined. However, we did show here that megakaryocytic specific gene expression can be induced following Fli-1 transduction in erythroleukemia cells, and can be further elevated by treatment with PKCAs. Based on these observations, we propose that Fli-1 expression is necessary for erythroleukemic cells to di-differentiate into cells resembling megakaryocyte/erythrocyte progenitors (MEP) [Vecchiarelli-Federico et al, unpublished results]. Subsequent events induced by the PKCA compounds, including Serine/Threonine phosphorylation of Fli-1 may accelerate differentiation of these MEPs toward megakaryocytes - a process we termed erythroid-to-megakaryocytic conversion (EMC).

Protein-protein interaction between Fli-1 and GATA-1 was recognized as a critical event during megakaryocytic differentiation [41, 51]. Indeed, at low concentrations of PKCAs downregulated GATA1, at higher concentration they significantly upregulated GATA1 expression in erythroleukemia cell lines expressing Fli-1. However, these compounds drastically downregulated GATA1 in Fli-1-non-expressing K562 cells. Expression of Fli-1 in K562 was shown to moderate this downregulation, highlighting the importance of GATA1 expression in megakaryocytic differentiation. This finding is consistent with the formation of a heptad including five key hematopoietic transcription factors: FLI-1, GATA1, GATA2, RUNX1 and TAL1/SCL during megakaryopoiesis [41, 51]. Our data suggest that Fli-1 is a major player in this complex interaction and that its downregulation or activation below or above certain thresholds can alter the fate of progenitor cells to undergo either erythroid or to megakaryocytic differentiation, respectively (see model, Figure 11F).

We show that transcriptional activation of Fli-1 by the PKCAs is induced by MAPK/ERK activation (Figure 8G). However, inhibition of MAPK/ERK with a specific inhibitor only had a marginal effect on growth repression induced by these compounds, implicating other pathways induced by PKC or by these drugs. Indeed, the cell attachment induced by the PKCA compounds was mediated by PKC independently of MAPK/ERK. In addition, in Fli-1 negative K562 cells, the PKCA compounds induced apoptosis in the absence of differentiation. Thus, induction of apoptosis by these compounds may be mediated by an alternative pathway or another member of the PKC family.

The PKCA compounds exhibited potent inhibitory potential when administered into a mouse model of leukemia. The level of tumor inhibition *in vivo* correlated with the ability of the drug to activate PKC *in vitro*. This anti-leukemic activity was associated with EMD in the spleens of leukemic mice. Thus, paradoxically, activation of PKC, despite unambiguous tumor promoting function in certain contexts, can be beneficial for the treatment of Fli-1-induced leukemia. Importantly, inactivating mutations in PKC occur frequently in diverse cancer types and correction of these mutations by CRISPR-mediated genome editing can block tumor progression [52]. Thus, PKC isozymes may function as tumor suppressors in certain other contexts as described herein.

In summary, by screening a library of compounds from natural products, we identified several phorbol ester-like agents with strong anti-leukemic activity. These compounds were shown to exert their anti-leukemic activity in part through activation of Fli-1. Mechanistically, they modulate Fli-1 by activating PKC and MAPK, leading to phosphorylation and activation of this TF. The PKCA-mediated activations of Fli-1 was shown to induce an erythroid-to-megakaryocytic differentiation (EMD) in cultured cells and in a leukemic model *in vivo* and suppress tumorigenesis. Overall, these data suggest that both activation and inactivation of Fli-1 may have therapeutic benefit for the treatment of leukemia and likely other type of cancers expressing this ETS member.

## MATERIALS AND METHODS

### Cell lines

Murine Friend virus-induced erythroleukemic cell lines CB7, human erythroleukemic cell lines K562 and HEL, human embryonic kidney HEK293T cell lines were maintained in Dulbecco's Modified Eagle Medium supplemented with 5% fetal bovine serum (HyClone, GE Healthcare, Australia).

### Tumor induction and *in vivo* drug studies

Viral supernatants from NIH-3T3 cells transduced with F-MuLV clone 57 plasmid were harvested and frozen at -80°C. New born mice were inoculated by intraperitoneal F-MuLV injections, as described [23]. Five weeks post infection, leukemic mice were injected IP, every other day for a total of six injections with A75 and A89 compounds [3 mg / kg of bodyweight] or DMSO as vehicle control, and monitored for signs of disease. Mice showing the signs of late stage disease were sacrificed and survival was determined, as described [23].

### Cell cycle and apoptosis analysis

For apoptosis and cell cycle analysis, erythroleukemia cell lines were incubated with compounds or DMSO as a vehicle control for 72 hours; after that cells were washed by cold PBS. For apoptosis experiment, cells were stained by Annexin V and PI apoptosis detection Kit (BD Biosciences, Franklin lakes, NJ) following the kit guidelines and analysed by flow cytometer. For cell cycle analysis, cells were fixed by cold 75% ethanol overnight at -20°C. After washed by cold PBS, cells were stained in PI for 40 mins at 37°C, then analysed by flow cytometer.

### Flow cytometric analysis

Immunofluorescence staining was performed to determine expression of various molecules on the cell lines, tumors cells of drug-treated and control (DMSO–treated) mice, as described [53]. In brief, 10^6^ cells were incubated with CD16/CD32 blocking antibody or human Fc receptor binding inhibitor (eBioscience, San Diego, CA) for 20 min, then stained with primary antibodies for 1h on ice. Primary antibodies were as follows: Phycoerythrin-conjugated anti–mouse or anti-human CD41, CD61 and Allophycocyanin-conjugated anti-mouse CD71 (eBioscience). Then cells were washed and resuspended in 500 μL of PBS Phosphate Buffered Saline. A total of 10^4^ events were collected using the FACSCalibur flow cytometer (BD Biosciences) and analysed using CellQuest Pro software (BD Biosciences).

### Drug screening and luciferase assay

A library of 1500 compounds, isolated from medicinal plants in China, was used to screen for anti-Fli-1 activity, as previously described [23]. In brief, HEK293T cells were co-transfected with FB-Luc (1.25μg) and MigR1 (1.25μg) or MigR1-Fli-1 (1.25μg) expression vector for 24 hours using Lipofectamine 2000 (Life Technology, Beijing, China) following the manufacturer's protocol. The transfected cells were plated into 96 well plates, incubated for 12 hours and then treated with compounds (5μM) or other indicated compounds for additional 12 hours. Luciferase activity was determined, as described [23, 54].

### Western blotting, immunoprecipitation and inhibitory compounds

Western blotting was conducted as described [23]. Polyclonal rabbit antibody for Fli-1, PKCδ, Phospho-PKCδ, PKCα, Phospho-PKCα were obtained from Abcam (Abcam, Cambridge, UK); ERK, phospho-ERK and GATA1 antibodies from Cell Signalling Technology (CST, Danvers, MA01923), GAPDH from Sigma (Sigma Aldrich, China) and β-actin from Proto-Technology (Protein-Tech, Bucuresti, Romania). The dilution of antibodies was used according to the manufacturer instructions. The inhibitors of PKC (GO6983), MEK (U0126), RAF (Sorafenib Tosylate), JNK (SB600125), Calcimycin, Rottlerin, and P38 (SB203580) were all obtained from Sellectchem (Sellectchem, Houston, USA). These compounds were dissolved in DMSO and added to the cells at indicated concentration, as described in the results section.

For immunoprecipitation, Extracts isolated from HEL and CB7 cells were immunoprecipitated using anti-Fli-1 antibody followed by western blot with anti-phospho Serine/Threonine antibody (Abcam), as previously described [23].

### Quantitative real-time PCR

RNA levels were determined by quantitative Real-time PCR in a StepOne Plus thermal cycler (Applied Biosystems, Forest city, Ca) using specific primers and SYBR® select Master Mix (Life technologies, Carlsbad, USA) as described [54]. The β-actin gene was used as control, the list of primers used for RT-PCR and Q-RT-PCR is as follow: All experiments were performed in triplicates and repeated at least two times. The human β-actin primers: 5′ forward primer (5′-CATGTACGTTGCTATCCAGGC-3′) and 3′ reverse primer (5′-CTCCTTAATGTCACGCACGAT-3′). The murine β-actin primers: 5′ forward primer (5′-GTGACGTTGACATCCGTAAAGA-3′) and 3′ reverse primer (5′-GCCGGACTCATCGTACTCC-3′). The human/murine Fli-1 primers: 5′ forward primer (5′CCACACT GTGGACACAGGAG-3′) and 3′ reverse primer (5′-TCG GTGTGGGAGGTTGTATT-3′). The human PF4 primers: 5′ forward primer (5′- TGCAGTGCCTGT GTGTGAAGAC-3′) and 3′ reverse primer (5′- CTTCCATTCTTCAGCGTGGCTATCA -3′). The human GP6 primers: 5′ forward primer (5′-CACAGGAACCTC TGTGACCC-3′) and 3′ reverse primer (5′-GGACCAGC TGGAGAGTCTGA-3′). The human GATA-1 primers: 5′ forward primer (5′-TGGTGGCTTTATGGTGGTG-3′) and 3′ reverse primer (5′-CCTTGGTAGAGATGGGCAGT-3′). The human β-globin primers: 5′ forward primer (5′-GCAACCTCAAACAGACACCA-3′) and 3′ reverse primer (5′-CCTCACCACCAACTTCATCC-3′). The human EKLF primers: 5′ forward primer (5′-GGTTGC GGCAAGAGCTACA-3′) and 3′ reverse primer (5′-GTCAGAGCGCGAAAAAGCAC-3′). The human RUNX1 primers: 5′ forward primer (5′-CTACCAATA CCTGGGATCCAT-3′) and 3′ reverse primer (5′-GAAAGT TCTGCAGAGAGGGTT-3′).

### Cloning and viral infection

The Fli-1 inducible expression construct (PInducer20-Fli-1) was generated by cloning Fli-1 cDNA into the Sal1-Xho1 sites of PInducer20 plasmid (Addgene, Cambridge, USA) [55]. For lentivirus production, PInducer20-Fli-1 and packaging plasmids psPAX2 and PMD2.D (a gift from Didier Trono, Addgene plasmid # 12259 and 12260) were co-transfected into HEK293T cells, as described [55]. 48 hours post transfection, the supernatants were harvested, the viruses were centrifuged at 2500 x g for 10 min, and filtered through 0.45 μm filters. The viruses were either used for infection freshly, or stored at -80°C freezer. For leukemic cells infection, K562 cells were cultured and grown in the presence of fresh supernatant of lentivirus producing cells. After 24 hours infection, medium was changed and cells were grown in presence of Neomycin (Solanbio, Beijing, China), until G418 -resistant cells were obtained.

### Survival and statistical analysis

Mice survival rates were computed and plotted according to the nonparametric Kaplan-Meier analysis. Statistical analysis was performed using the two-tailed Student's t-test with significance considered at P<0.05, and by analysis of variance using Origin 3.5 software (Microcal Software, Northampton, MA, USA).

### Animal care

Animal care is in accordance with the guidelines of the Guizhou Medical University and China Council of Animal Care.
